# Upconverting Nanoparticles as a New Bio-Imaging Strategy—Investigating Intracellular Trafficking of Endogenous Processes in Neural Tissue

**DOI:** 10.3390/ijms24021122

**Published:** 2023-01-06

**Authors:** Karolina Zajdel, Justyna Janowska, Małgorzata Frontczak-Baniewicz, Joanna Sypecka, Bożena Sikora

**Affiliations:** 1 Mossakowski Medical Research Institute, Polish Academy of Sciences, 5 Pawinskiego Str., 02-106 Warsaw, Poland; 2 Institute of Physics, Polish Academy of Sciences, Al. Lotników 32/46, 02-668 Warsaw, Poland

**Keywords:** upconversion, organotypic hippocampal slice cultures (OHSCs), nano-bio interactions, cytotoxicity, cellular uptake pathways

## Abstract

In recent years, rare-earth-doped upconverting nanoparticles (UCNPs) have been widely used in different life sciences due to their unique properties. Nanoparticles have become a multifunctional and promising new approach to neurobiological disorders and have shown extraordinary application potential to overcome the problems related to conventional treatment strategies. This study evaluated the internalization mechanisms, bio-distribution, and neurotoxicity of NaYF_4_:20%Yb^3+^,2%Er^3+^ UCNPs in rat organotypic hippocampal slices. TEM results showed that UCNPs were easily internalized by hippocampal cells and co-localized with selected organelles inside neurons and astrocytes. Moreover, the UCNPs were taken into the neurons via clathrin- and caveolae-mediated endocytosis. Propidium iodide staining and TEM analysis did not confirm the adverse effects of UCNPs on hippocampal slice viability and morphology. Therefore, UCNPs may be a potent tool for bio-imaging and testing new therapeutic strategies for brain diseases in the future.

## 1. Introduction

The dynamic development of nanotechnology created opportunities to apply nanomaterials application in various fields of science. Nanomaterials’ ability to penetrate the cell membrane and manipulate their physicochemical properties holds great promise for improving the efficacy of therapy or bio-imaging. It brings the opportunity to obtain revolutionary solutions in medicine, including cancer treatment and promising markers used for in vitro and in vivo bio-imaging. Understanding the interactions of nanoparticles (NPs) with biological systems is fundamental to developing novel nanosystems. Despite extensive research in this field, the amount of information on the impact of new nanomaterials on human health is still disproportionate to the rate of development of this discipline. Due to the broad spectrum of the desirable properties of nanomaterials and their potential application areas, their safety assessment is fundamental to the rational design of new functional nanomaterials. There is an urgent need for nanomaterials characterized by low cytotoxicity and high biocompatibility.

An important issue is that depending on the application, stable or easily degradable NPs are preferred. NPs with the function of releasing ions, such as silver NPs (AgNPs) in the antimicrobial product [1], or iron oxide NPs in the treatment of anemia [2], should be biodegradable. On the other hand, NPs used as contrast agents (e.g., iron and gadolinium oxide NPs in MRI, AuNPs and AgNPs in surface plasmon resonance) or for bio-imaging (e.g., quantum dots, QDs, or up-converting nanoparticles, UCNPs) should be quickly and thoroughly removed from the body, without tissue accumulation effects. A better understanding of the interactions between the NPs and cells is essential for biomedical applications and the safety issues of nanomaterials. To develop safe and efficient nanosystems for biology and medicine applications, many factors need to be considered: (1) the physicochemical properties of NPs (e.g., size, shape, surface charge, and surface chemistry) [3]; (2) colloidal stability in solutions [4]; (3) degradation or removal rate [5]; (4) biocompatibility [6]; (5) bioaccumulation [7]; (6) the route of administration (e.g., intravenous, oral, nasal and pulmonary, transdermal); and (7) the type of target cell or tissue [8,9,10].

The ability of NPs to cross various biological barriers, including blood–brain barriers (BBB), has prompted research into their use in the diagnosis and treatment of central nervous system (CNS) diseases [11,12,13,14,15]. The importance of nanoscience in the treatment of diseases of the nervous system covers a growing number of issues [16,17]. Although this is a relatively poorly studied field, there are extensive attempts to use nanomaterials as drug carriers [18], develop strategies in neuroprotection research, scaffolds in neurodegeneration research, as well as for neuro-imaging, or tools for neuro-operation [19]. The main nanotechnology approaches for neurodevelopmental, neurological, and neuropsychiatric disorders include: (1) the use of NPs or nanocarriers for drug delivery or gene therapy; (2) the use of nanotechnology to reconstruct, reinforce, and stabilize the cytoskeleton matrix; (3) fabrication of bio-hybrid devices for transporting various compounds; and (4) coating electrodes with NPs [20]. Nowadays, nanoscale drug design and delivery are of great interest. Neuronal damage in many CNS disorders has favored the search for new drugs, gene therapies, and the creation of advanced prosthetic nanodevices [20]. Recent progress in nanotechnology-based theranostic is expected to advance precision and personalized CNS medicine [16]. Currently, among the most popular NPs used in bio-medicine, a new generation of NPs based on rare-earth elements deserves special attention. The rare-earth-doped oxides or fluoride NPs with upconverting properties (UCNPs) have been studied intensively [21,22,23,24,25]. Typically, UCNPs are simultaneously doped with sensitizing rare-earth ions, e.g., Yb^3+^ and Er^3+^, Tm^3+^ or Ho^3+^ [26,27,28]. Sensitizing rare-earth ions absorb low-energy radiation in the near-infrared (NIR) region and then non-radioactively transfer this energy to the long-lived excited metastable electronic states of activating rare-earth ions [29]. The absorption of subsequent low-energy NIR photons and energy transfer processes (ETU) promote rare-earth-activating ions to their higher electronic excited states, thus leading to the radiation of high-energy photons in the ultraviolet, visible, or NIR spectral ranges [30,31].

UCNPs are a better alternative to traditional optical imaging due to several advantages, such as resistance to photobleaching, low background autofluorescence in biological material, deep tissue penetration, and minimal photodamage [32,33,34,35,36]. These characteristics make UCNPs potentially applicable in biology and medicine [37], especially in the in vitro and in vivo bio-imaging [36,38,39], photodynamic therapy (PDT) [40,41], or photothermal therapy (PTT) [42].

Among the many attempts to use various types of NPs for applications in neuroscience, UCNPs have been used for imaging and cancer therapy [43], optogenetic neuronal control [44,45], as biosensors for the detection of Zn^2+^ ions in mouse brain slices with Alzheimer’s disease, and zebrafish [46], deep tissue optical stimulation, and the imaging of mouse brain slices [47], or have been used as the ion channel blocker of neurons in living brain slices [48]. Recently, most studies have been concerned with trichromatic UCNPs in in vivo optogenetic mouse neurons [49].

Organotypic brain slice cultures are a valuable tool for studying the physiological and pharmacological processes of substances in tissues, the cellular and molecular mechanisms underlying CNS disorders and evaluating potential treatments of CNS diseases [50]. Using tissue models such as tissue explants (slices) seems an excellent intermediate step between cell culture and living organism tests. In addition, the advantage of using brain slice models for in vitro studies is that they preserve the cytoarchitecture and microenvironment of tissues in vivo. This model allows for observing the changes caused by nanomaterials in tissues and testing new therapeutic strategies in vitro. The experimental model provides information on the processes under controlled conditions but is more advanced than commonly used cell lines or primary neuronal cultures. The brain area that is most widely cultured in vitro is the hippocampus [50]. The organotypic hippocampal slice cultures (OHSCs) model comprehensively studies the interactions between neurons, macroglia (astrocytes, oligodendrocytes), microglia, and vasculature. It is often used to study neuronal damage, synaptic plasticity, cell proliferation, and maturation. Understanding the mechanisms of NPs internalization and their impact on tissue morphology is crucial for developing new cell therapies for diseases that can be modeled using hippocampal tissue. Designing and obtaining NPs for drug delivery to the brain in a controlled way could improve the effectiveness of brain therapies [50,51].

This study aimed to evaluate the biological interactions between the NaYF_4_:20%Yb^3+^,2%Er^3+^ UCNPs and ex vivo model of OHSCs, including:

- The determination of the possibility of NaYF_4_:20%Yb^3+^,2%Er^3+^ UCNPs internalization
- Cytotoxicity evaluation and ultrastructural changes analysis after exposure to NaYF_4_:20%Yb^3+^,2%Er^3+^ UCNPs

## 2. Results

### 2.1. Physicochemical Properties of the β-NaYF_4_:20%Yb^3+^,2%Er^3+^ UCNPs

**Figure 1:**
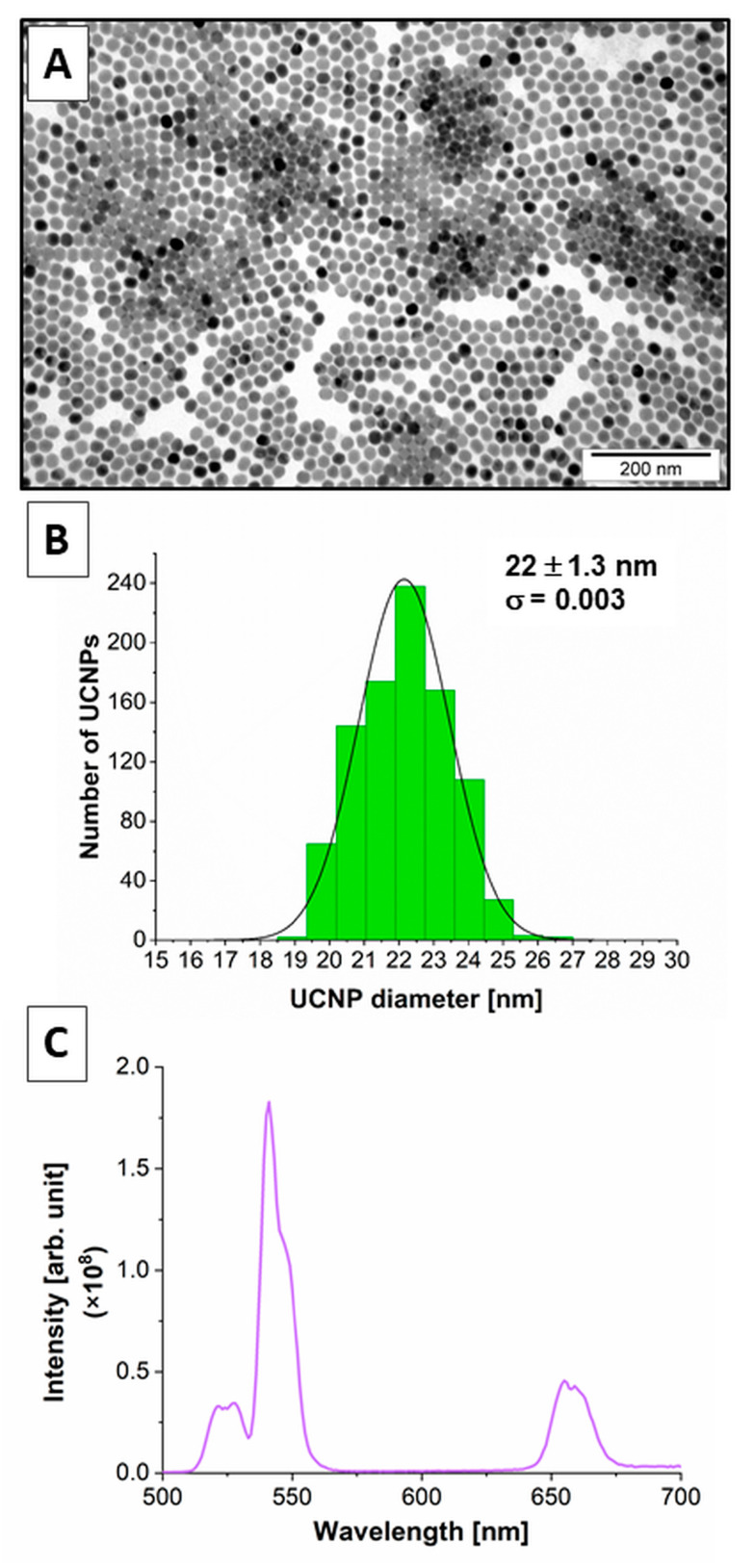
(**A**) TEM and (**B**) Size distribution histogram for β-NaYF_4_:20%Yb^3+^,2%Er^3+^ UCNPs. (**C**) Upconversion luminescence spectra of β-NaYF_4_:20%Yb^3+^,2%Er^3+^ UCNPs solution in cyclohexane at a 1 mg mL^−1^ concentration after 980 nm continuous wave laser excitation with a power density of 7.4 W cm^−2^.

The morphology and size of the oleic acid-capped and ligand-free β-NaYF_4_:20%Yb^3+^,2%Er^3+^ UCNPs were measured by TEM (Figure 1A,B). Several images were used to obtain a total of N = 931 particle sizes. Subsequently, a particle-size histogram was mounted using the Sturges method, as described before [52]. The bin-width (W) was obtained from the relation: W = (D_max_ − D_min_)/k, where k = 1 + 3.322 log(N). For our NPs, k was 10, and W was 0.85. The histogram was modeled by a normal distribution, as shown in Figure 1B. A mean particle size of 22 nm was estimated by using the relation = D_0_exp(σ^2^/2), where D_0_ is the size and σ is the polydispersity parameter (PDI). The PDI was obtained based on [53] and was 0.003. That means that the UCNPs were very homogeneous and had a regular hexagonal structure.

Oleic acid was effectively released from the UCNPs to obtain the hydrophilic UCNPs with good dispersibility in aqueous solutions for biological applications. The EDS and XRD methods were used to confirm the elemental composition of the UCNPs and the hexagonal structure of UCNPs (the results have been previously published) [54,55].

The luminescence emission spectra after 980 nm laser excitation of β-NaYF_4_:20%Yb^3+^,2%Er^3+^ UCNPs are characterized by high-efficiency upconversion to the green (~520 nm and ~540 nm) and red (~650 nm) light (Figure 1C). The mechanism of upconversion was discussed before [39,54,55,56].

### 2.2. TEM Results and Analysis

**Figure 2:**
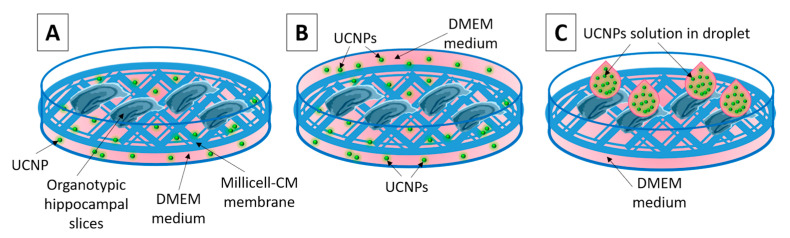
Diagram showing the three analyzed methods of applying UCNPs to the OHSC: (**A**) UCNPs are added to 1 mL of the culture medium under the membrane, (**B**) the OHSCs placed on the membrane are immersed in the culture medium containing UCNPs, (**C**) UCNPs solution in droplet applied to the slice surface.

TEM analysis of the hippocampal cells was performed. The presence of UCNPs was tested in three ways of UCNPs administration in OHSCs (Figure 2):

(1) The UCNPs were added to 1 mL of medium (0.5; 1; 5 and 10 μg mL^−1^, 24 h incubation);
(2) The UCNPs were added to 2 mL of medium with immersed slices (0.5; 1; 5 and 10 μg mL^−1^, 1 h incubation);
(3) The UCNPs were applied in droplets directly to the top of the slice surface (0.5; 1; 5 and 10 μg 4μL^−1^, 2 h incubation).

**Figure 3:**
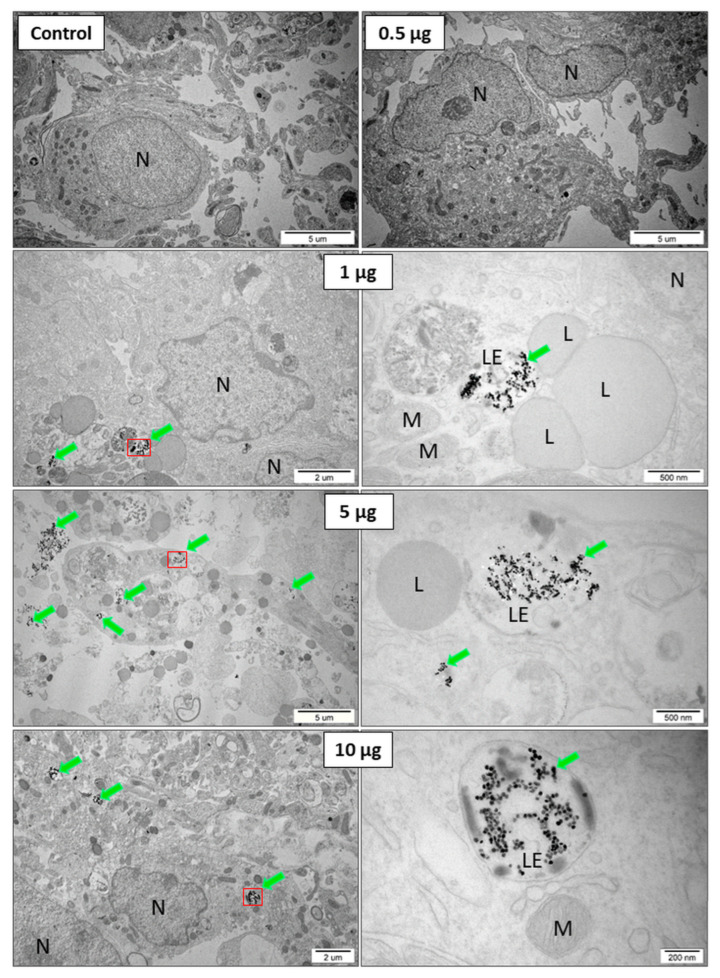
Variant 3. The OHSCs, after 2 h of incubation without UCNPs (control) and with 0.5; 1; 5, and 10 μg 4 μL^−1^ of UCNPs applied as droplets on the surface of sliced placed on the membrane. Aggregates of UCNPs are marked with green arrows. UCNPs inside endosomes are marked with a green arrow and a red outline (at a higher magnification next to it), N—nucleus; L—lysosome; M—mitochondria; and LE—late endosomes.

In variant 1, the presence of UCNPs was observed in hippocampal cells only at very high concentrations, i.e., 10 μg mL^−1^ (Figure S1). In variant 2, no UCNPs were found inside the cells. Ultrastructural analysis of the OHSCs showed the presence of large clusters of UCNPs only in the extracellular space at the highest concentration (10 μg mL^−1^) (Figure S2). Based on the obtained results, it was concluded that these two methods of introducing UCNPs to the slices were ineffective. However, in variant 3, i.e., for the application of UCNPs directly on the slices, the effective internalization of the UCNPs was observed in the range of all of the tested concentrations. The UCNPs inside endosomes are marked with a green arrow in Figure 3.

**Figure 4:**
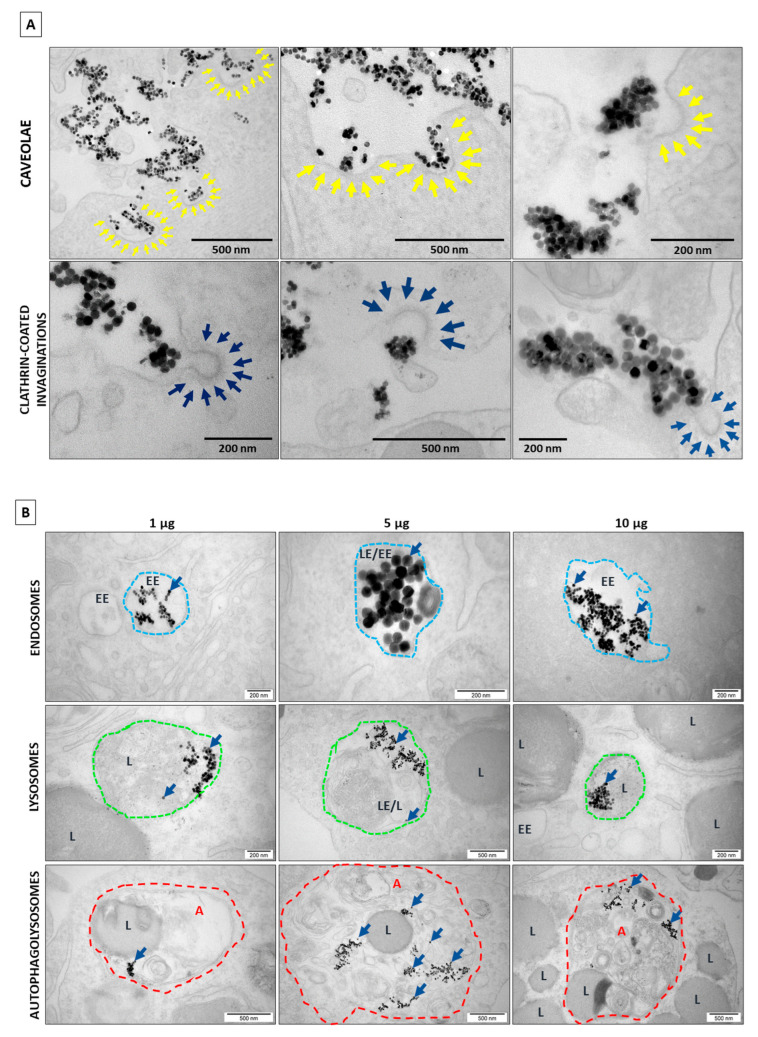
(**A**) The formation of the caveolae (marked with yellow arrows) and clathrin-coated vesicles (marked with dark blue arrows) at the cell membrane in the presence of UCNPs after 2 h of incubation (a droplet contains 1 μg of UCNPs). (**B**) The co-localization of UCNPs (marked with dark blue arrows) with various intracellular structures. UCNPs are administered in a droplet on the top of the slice after 2 h of incubation, enclosed in early endosomes (EE—outlined in blue dashed line), in late endosomes (LE) or lysosomes (L—outlined in green dashed line), in autophagolysosomes (A—outlined in red dashed line).

In the next stage of the experiment, the morphology and ultrastructural features of the cell organelles were assessed after the exposure of the OHSCs to the UCNPs in variant 3, depending on the concentrations. The presence of UCNPs generally was observed in neurons. Numerous cavities in the cell membrane of neurons at the points of contact with the UCNPs were visualized (arrows in Figure 4A). The presence of large UCNP aggregates inside the endosomes in the neurons was observed. The UCNPs were found in early and late endosomes, lysosomes, and large autophagolysosomes (blue arrows in Figure 4B). Similar to HeLa and 4T1 cells [54,55], the UCNPs in the cytoplasm, other intracellular structures or the cell nucleus were not observed. At higher concentrations of UCNPs (10 μg), an increased number of lysosomes was observed in neurons. This suggests that the UCNPs at high concentrations may induce autophagy processes in the cells. The observations showed that high concentrations of UCNPs did not affect the cell response. The UCNPs neither changed their localization in cells nor were released into the cytoplasm. The NaYF_4_:20%Yb^3+^,2%Er^3+^ UCNPs inside the hippocampal cells had stable behavior, and no changes in their morphology were observed.

### 2.3. The Confocal Microscope Imaging Results

**Figure 5:**
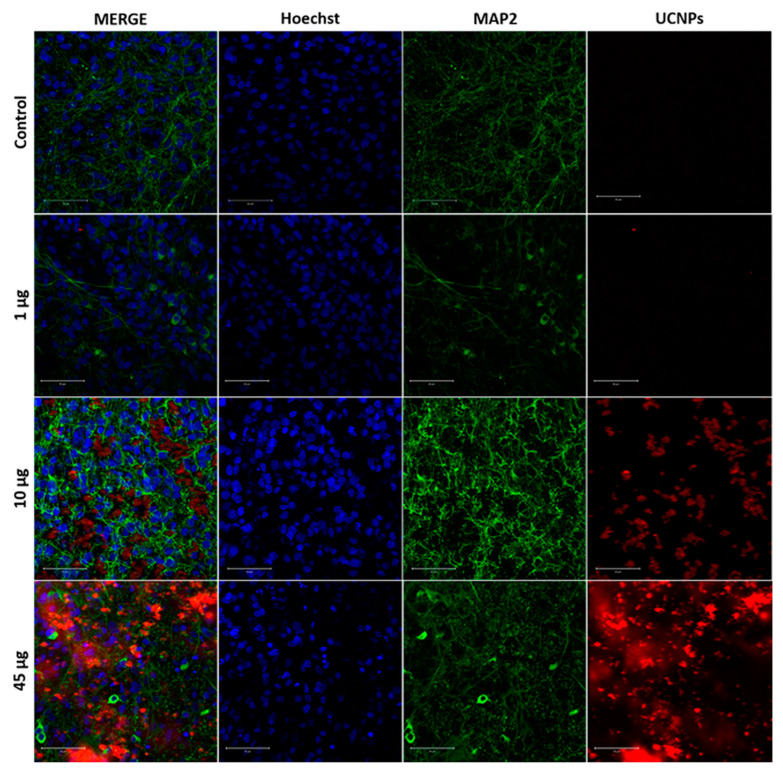
Confocal images of OHSCs after 2 h of incubation with UCNPs depend on the concentration (1, 10, and 45 µg in 4 µL of medium) applied in the form of droplets on the surface of the OHSCs. The UCNPs were excited with a 980 nm femtosecond laser (red color), neurons (MAP2) were excited with a 488 nm argon laser (green color), and cell nuclei were stained with Hoechst 33342 dye and excited with a 690 nm femtosecond laser (blue color). The presented MERGE image is a composite of all images. Controls were OHSCs without UCNPs. Scale bar—50 μm.

In further experiments, a confocal microscope analyzed the UCNPs’ penetration into the different types of cells of the OHSCs. The results confirmed the effective process of UCNP internalization by neurons and astrocytes and their spectroscopic properties. Figure 5 shows representative tricolor confocal images showing the presence of UCNPs in neurons after 2 h with a range of UCNP concentrations (1, 10, and 45 µg in 4 µL of medium). The amount of UCNPs increased with the UCNP concentration. The red color in Figure 5 indicates the UCNPs, the green color indicates the neurons, and the blue indicates the cell nuclei. The red color co-localized with green, which can be interpreted with some probability that UCNPs are inside neurons. The red channel showed only luminescence from the UCNPs. No autofluorescence was registered. This means that the UCNPs are a great candidate to be luminescence markers of biological structures.

The luminescence spectra of the UCNPs internalized by the neurons of the OHSC were recorded using the lambda scan mode (Figure S3). The emission spectra of the UCNPs inside the cells after excitation under the 980 nm infrared laser correlated with the emission spectra obtained from the spectroscopic analysis carried out during the physicochemical characteristics of UCNPs (Figure 1C). The results indicate the high efficiency of UCNPs luminescence emission in the OHSCs model. Moreover, the UCNPs channel (red color) exhibits a high signal-to-noise ratio with relatively high upconversion emission intensity. There was also no background autofluorescence. That allows for obtaining confocal images with excellent resolution and high contrast, which cannot be achieved with traditional one- or two-photon fluorescence imaging.

**Figure 6:**
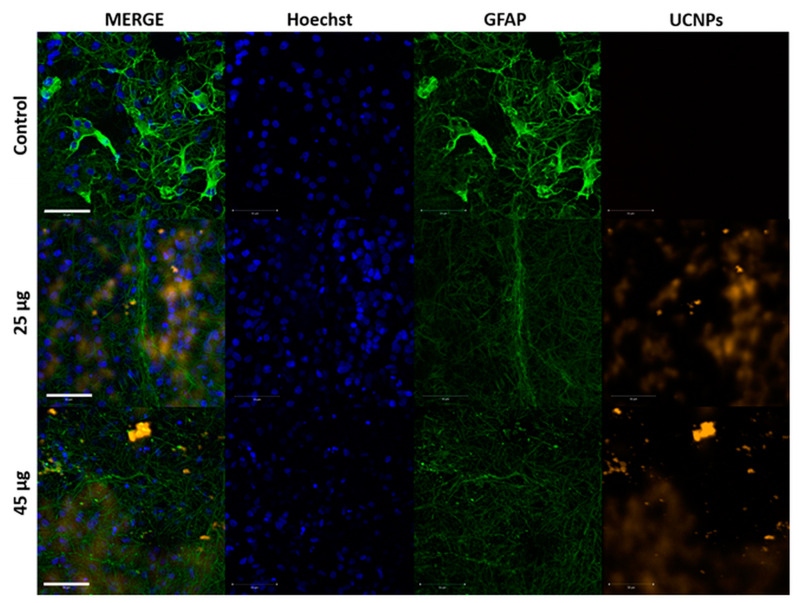
Confocal images of the OHSCs incubated for 2 h with 25 µg and 45 µg of UCNPs applied as droplets on the OHSCs surface. UCNPs were excited with a 980 nm femtosecond laser (orange color), astrocytes (GFAP) were excited with a 488 nm argon laser (green color), and cell nuclei were stained with Hoechst 33342 dye and excited with a 690 nm femtosecond laser (blue color). The presented MERGE image is a composite of all channel images. Controls were OHSCs without UCNPs. Scale bar—50 μm.

The presence of UCNPs was also confirmed inside astrocytes (GFAP), but in smaller amounts and at higher concentrations of 25 and 45 μg in 4 μL (Figure 6). To investigate the biodistribution of UCNPs in the cells of the OHSCs, an additional confocal microscopy thickness section analysis was performed, which consisted of a 3D reconstruction of the examined section in the Z-stack scanning mode (Figures S4 and S5). The Z-stack study performed on OHSCs incubated for 24 h with 1 µg and 10 µg of UCNPs showed that UCNPs were localized inside the cells at different levels in the organotypic slice.

### 2.4. Cell Viability after Exposure to UCNPs

In subsequent experiments, the viability of the hippocampal cells was examined by using Propidium iodide (PI) staining of the dead cells, and then fluorescent images were taken. The OHSCs were treated with UCNPs in different concentrations, added directly to the culture medium, or applied on the surface of the slices. The microscopic observations showed no tissue damage at all UCNP concentrations compared to untreated slices (Figure S6).

**Figure 7:**
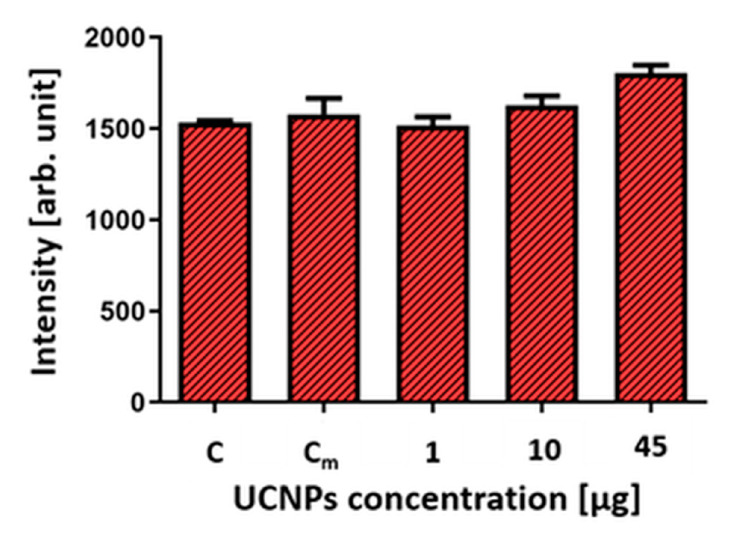
Effect of UCNPs on cell death of OHSCs. Each value is the mean obtained from four replicates for each variant. Statistical analysis was performed using one-way ANOVA with Tukey’s test, n = 4; *p* > 0.05. C—Control, the OHSCs untreated with UCNPs and medium; C_m_—control, OHSCs treated only with the medium.

Then, based on the results from fluorescence imaging, only variants with UCNPs applied as droplets were selected to determine the viability of the hippocampal cells. After the 24 h incubation, no statistically significant effect of UCNPs was observed on the cell viability of OHSCs compared to the control (without UCNPs). More substantial cell death in OHSCs exposed to 45 µg (45 µg in 4 µL) of the UCNPs was observed. It should be noted that the OHSCs model is a demanding model and highly sensitive to changes in cell culture conditions. This is evidenced by differences in the viability of control (C) OHSCs, untreated with UCNPs and medium, and OHSCs treated only with medium (C_m_), without UCNPs. The values in the graph are presented as the maximum PI fluorescence measured in ImageJ (mean grey value; arbitrary units) (Figure 7).

## 3. Discussion

To confirm UCNPs internalization by cell types other than HeLa, HEK293, 4T1, etc. [54,55,57], an electron microscopy analysis of the ex vivo culture of the organotypic hippocampal slices was performed. The effect of UCNPs on the OHSCs was investigated in several variants (UCNPs added to the culture medium or on the slice surface) and in different UCNP concentrations. In the variant with UCNPs in the medium, the internalization of UCNPs was observed only for high concentrations (above 10 μg mL^−1^). On the other hand, in the variant of direct UCNPs administration to the OHSC, the results indicated active internalization in all concentrations of UCNPs. After 24 h incubation of the OHSC with 10 μg UCNPs in a medium, the electron microscopy images showed a significant accumulation of large UCNP complexes that were not internalized by the cells. These difficulties seem to be associated with limited access to UCNPs from medium to slices. The presence of large complexes of UCNPs outside the cells could also be related to the short incubation time, so most UCNPs probably did not have time to interact with the cells. However, the UCNPs were found very close to the cell’s surface or between cells. This may suggest possible interactions with receptors on the cell membrane, eventually leading to UCNP internalization. It is further supported by numerous cell membrane invaginations formed by intracellular vesicles in direct contact with UCNPs. The variant directly applying UCNPs in a droplet on the OHSC surface was effective and efficient. The TEM results confirmed the presence of large clusters of UCNPs closed inside the vesicle structures present in neurons. The UCNPs were found in early and late endosomes, inside lysosomes, and in large autophagolysosomes. Similar to the in vitro model, as we showed before [54,55,57], the UCNPs in the cytoplasm of cells and the nucleus or inside other organelles were not observed. This indicates that the UCNPs were transported inside the cells by the endocytosis process. This was confirmed by numerous intracellular vesicles and endosomes containing UCNPs and by folded cell membranes with numerous invaginations at the contact areas with the UCNPs. The formation of such numerous invaginations and membranes’ behavior in many cells was not observed in brain slices not treated with UCNPs.

The internalization of UCNPs was probably proceeded by clathrin- and caveolae-mediated endocytosis. This is evidenced by plasma membrane invaginations called caveolae and plasma invaginations with a characteristic coat of clathrin around the endocytosed vesicles.

The TEM observations have shown that the NaYF_4_:2%Er^3+^,20%Yb^3+^ UCNPs are easily internalized by hippocampal cells but only under technically unlimited access to the slices. The results suggest that OHSCs may become an effective model for testing various therapeutic strategies for neurological diseases using nanostructures. High expectations for brain imaging capabilities using UCNPs application are attributed to their unique upconverting properties. Due to the upconversion of UCNPs, imaging depth in living brain tissue may be possible. In addition, the tested material (the embedded fragment of the slice in an epoxy resin) was trimmed several times to confirm the UCNP presence and distribution in the deeper areas of the slice sections. A single TEM electronogram of cells is a single cross-section with a thickness of approx. 40–60 nm from tissue fragment with a total thickness of 400 μm. It allowed us to conclude that the UCNPs (in very high concentrations) are not only dynamically internalized by cells but also transported to their other slice regions. Moreover, the large agglomerates of non-internalized UCNPs accumulated between the spaces around the cells. No electron-microscopic studies are available in the literature with the in-depth assessment morphology of OHSCs exposed to nanomaterials. There is no information about the intracellular localization of NPs in slices. Few studies confirmed the presence of NPs in the OHSCs by TEM. These studies concerned QD biofunctionalized with the Paml1 peptide (CL4 QD-Palm1), bound explicitly to neurons [58]. Other studies showed the neuroprotective mechanism of CeO_2_ NPs in a mouse hippocampal brain slice model of cerebral ischemia [59]. Another group showed a distribution of pure uncoated AgNPs in the cytoplasm of the neurons (isolated rat hippocampus cells) [60].

Our research on the distribution of UCNPs in the culture of the organotypic hippocampal slices showed that the ability to penetrate hippocampal cells and the efficient uptake of the UCNPs are natural features of UCNPs to enter cells and accumulate in selected intracellular organelles quickly. Analysis based on the reconstruction of the tissue cross-section allows for obtaining much more observations and results than imaging with other methods (e.g., confocal microscopy), which means that imaging using TEM is a crucial technique and the gold standard in the microscopic analysis of nanosized materials.

In the next stage of the experiment, the morphology and ultrastructural features of the cellular elements of the OHSCs after exposure to the UCNPs, depending on the concentrations, were determined by TEM analysis. The UCNPs inside the hippocampal cells were not toxic in the range of the tested concentrations and exposure times. It should also be taken into account that the OHSC model is highly demanding and very sensitive to any changes in vitro. Therefore, due to numerous technical difficulties and to keep the organotypic slices in good condition for the experiment time, it was decided to perform analyses only on slices cultured for up to 7 days and incubation with UCNPs no longer than 24 h. The attempts of prolonged exposure of OHSC to UCNPs, performed on slices cultured up to 14 days and after 48 h of exposure, including the control variants (without UCNPs), were impossible to evaluate due to the unsatisfactory quality of OHSC. All of the experimental variants were performed in at least three replications.

At higher concentrations of UCNPs, an increased number of lysosomes and autophagolysosomes was observed, which probably suggests that the presence of UCNPs could induce autophagy in neurons. This is particularly interesting because many studies show that the pharmacological modulation of autophagy is neuroprotective in models of many diseases [61]. Recently, Phiwchai et al. showed that ferric–tannic nanoparticles could induce autophagy in neurons and promote cellular clearance in the neurodegenerative processes [62]. On the other hand, C60 fullerene nanoparticles could inhibit upregulated autophagy and neurodegenerative processes in the hippocampus of diabetic rats [63].

Based on the presented results, it can be concluded that UCNPs are easily internalized by hippocampal cells. The bio-distribution of UCNPs inside the cells was closely related to the dynamics of processes inside the cell and the predisposition of UCNPs to localize only with specific intracellular organelles, such as endosomes and lysosomes. This model may be beneficial for studying the biodistribution of UCNPs in tissue and assessing morphological and ultrastructural changes caused by the presence of nanomaterials. Furthermore, due to technical limitations and a short period of keeping slices in good condition, it seems highly justified to plan further experiments only with bio-functionalized UCNPs. Targeted cell therapies for nervous system diseases with bio-functional UCNPs would have a more positive effect. At this point, research on non-functional UCNPs has been completed.

The results showed that each analysis stage should include detailed studies to eliminate the risk of nanomaterials toxicity, especially in clinical applications. NPs should be carefully characterized and studied in detail in biological systems at every experimental design stage. At the same time, the obtained results are a beautiful aspect of ex vivo biological imaging of tissue fragments using the unique upconverting properties of UCNPs.

## 4. Materials and Methods

### 4.1. Synthesis of β-NaYF_4_:20%Yb^3+^,2%Er^3+^ UCNPs

The synthesis of oleic acid-coated and ligand-free β-NaYF_4_:20%Yb^3+^,2%Er^3+^ UCNPs was described previously [54].

### 4.2. Physicochemical Characteristics of β-NaYF_4_:20%Yb^3+^,2%Er^3+^ UCNPs

The morphology and structure of the β-NaYF_4_:20%Yb^3+^,2%Er^3+^ UCNPs were characterized using JEOL JEM-1011 TEM Japan with an operating voltage of 80 kV.

The photoluminescence (PL) spectra of the β-NaYF_4_:20%Yb^3+^,2%Er^3+^ UCNPs were measured using a spectrophotometer (Horiba Jobin Yvon Fluorolog3). A sample of UCNPs was excited with a NIR diode laser (LU0980D300-DNA014, Lumics, Berlin, Germany) with a wavelength of 980 nm (CW). The PL of the upconversion spectra was measured in the visible range from 500 nm to 700 nm at room temperature. A laser power density of 980 nm was 7.4 Wcm^−2^.

### 4.3. Ex Vivo Culture of Organotypic Hippocampal Slices (OHSCs)

The hippocampi used in the experiments were isolated from 7-day-old Wistar rat pups, according to the procedure previously described by Sypecka and Sarnowska [64]. The isolation process was approved by IV Local Ethics Committee on Animal Care (decision no. 39/2015) and used as indicated by the Ministry of Science and Higher Education.

**Figure 8:**
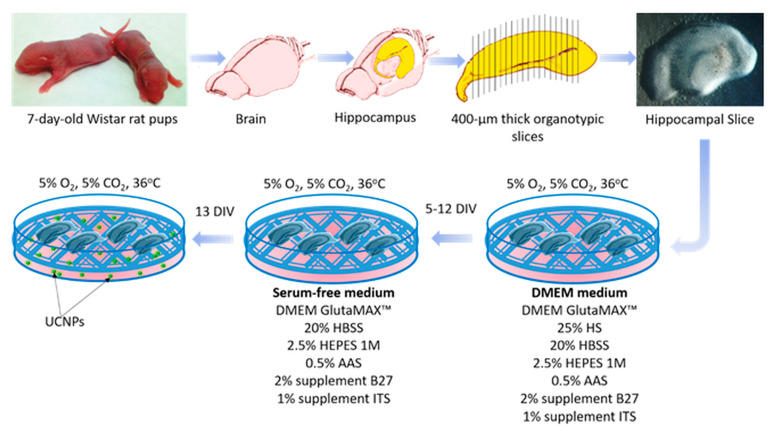
Schematic representation of the experimental design.

Briefly, after decapitation, the brain hemispheres were removed, and the hippocampi were isolated and cut crosswise into 400-µm-thick sections using a McIlwain Tissue Chopper (Campden Instruments, Loughborough, UK). The slices were pre-selected under a stereoscopic microscope (Opta-Tech, Warsaw, Poland). The slices with a preserved structure of the CA1 and CA2 regions were selected for the experiments. The four cut sections were placed on a Millicell^®^ Cell Culture Inserts membrane (Merck Millipore, Burlington, MA, USA) with a pore diameter of 0.45 µm and placed in 6-well plates (Thermo Scientific, Waltham, MA, USA). In each well was 1 mL DMEM GlutaMAX™ culture medium with high glucose content, supplemented with 25% inactivated horse serum (HS), 20% Hank’s Balanced Salt Solution (HBSS), 2.5% (4-(2-hydroxyethyl)-1-piperazineethanesulfonic acid (HEPES) (1 M), 0.5% Antibiotic Antimycotic Solution (AAS) (10,000 U/mL penicillin, 10 mg mL^−1^ streptomycin and 25 μg mL^−1^ amphotericin B), 2% B-27 supplement, and 1% Insulin–Transferrin–Selenium (ITS) supplement. The culture was carried out under physiological normoxic conditions to better reflect the neural tissue environment, i.e., in the presence of 5% O_2_ and 5% CO_2_, at a temperature of 36 °C and with humidity of 90%. The medium was changed every 2–3 days. During the first 4 days, the slices were kept in a medium with supplements. Next, from the 5th day in vitro (DIV) onwards, the serum concentration in the culture medium was gradually decreased by 6.25% for the following 7 days. The UCNPs administration began on the 13 DIV (slices were kept in serum-free media) (Figure 8). 

### 4.4. UCNPs Administration to OHSC

Three variants were analyzed to develop an effective method of UCNPs administration in the OHSC (Figure 2).

(1) The method of adding the UCNPs solution directly to the medium (UCNPs should be internalized by cells from the medium) (Figure 2A).
(2) The method of immersion of OHSCs in the medium with UCNPs. Under these conditions, they were incubated for 1 h (Figure 2B).
(3) The method of the UCNPs solution applying by droplet (4 μL) directly to the surface of the slice and incubating for 2 or 24 h (Figure 2C).

### 4.5. TEM Analysis

TEM JEM-1011 was used for the detection of UCNPs as well as the observation of the ultrastructural changes in the hippocampal cells caused by the presence of UCNPs. The description is available in Supplementary Information.

### 4.6. Immunohistochemical Staining

Immunohistochemical analyses were performed to confirm the presence of UCNPs within the hippocampal cells. The immunohistochemical preparations were imaged using a 710 NLO confocal microscope (Carl Zeiss, Oberkochen, Germany) equipped with a femtosecond laser (Coherent Chameleon, Santa Clara, CA, USA). The description is available in the Supplementary Information and Table S1.

### 4.7. Cell Viability Analysis Using Propidium Iodide

To quantify the potentially toxic effects of the UCNPs on hippocampal cells, staining with fluorescent propidium iodide (PI) was performed. PI readily penetrates the cell membranes of dead or dying cells. The stained cells emit red fluorescence; thus, the higher the level of fluorescence, the greater the percentage of cells killed. The cytotoxic effect of UCNPs was determined by incubating the slices for 24 h with 1, 10, 45, and 50 μg of UCNPs in the form of droplets (the suspension of UCNPs in water was applied in 4 μL droplets) directly on the surface of the slice or to the culture medium (1 mL of medium). After incubation, the culture medium was withdrawn, and a fresh medium containing 2 µg mL^−1^ of PI was added to each well and incubated for 1 h at 36 °C. The fluorescent images were recorded under an Axio Vert inverted microscope (Carl Zeiss, Jena, Germany). The color images were converted to grayscale, and the mean fluorescence from each hippocampus slice was measured using ImageJ software.

### 4.8. Statistical Analysis 

The results were expressed as arithmetic means ± standard deviation (SD). Statistical analysis was performed using GraphPad Prism 9 software (GraphPad Software, San Diego, CA, USA) using the one-way ANOVA test with Tukey’s test or Dunnett’s test (for comparisons of more than two groups; 95% confidence interval, α = 0.05). The results were considered statistically significant for *p* < 0.05.

## 5. Conclusions

The internalization studies of the UCNPs were carried out in an ex vivo OHSC model that was temporarily incubated with different UCNP concentrations. As a result of the conducted analyses (TEM, confocal microscope), the active internalization of NaYF_4_:20%Yb^3+^,2%Er^3+^ UCNPs by hippocampal cells, their biodistribution (Z-stack analysis) and their accumulation in endosomes and lysosomes was confirmed. Toxicity studies (PI staining, TEM analysis) did not demonstrate a significant adverse effect on cell viability and ultrastructure.

The internalization of NaYF_4_:20%Yr^3+^,2%Er^3+^ UCNPs by hippocampal cells and their localization in specific cell structures indicate their possible use in neurotoxicity studies and their therapeutic potential for CNS disorders.

The NaYF_4_:20%Yb^3+^,2%Er^3+^ UCNPs exhibit effective luminescence in vitro, both in cells and tissues, making them functional infrared imaging nanosystems.

## Data Availability

Not applicable.

## Supplementary Materials

The following supporting information can be downloaded at: https://www.mdpi.com/article/10.3390/ijms24021122/s1.
