# Supplementary material for: Upconverting Nanoparticles as a New Bio-Imaging Strategy—Investigating Intracellular Trafficking of Endogenous Processes in Neural Tissue
Source: Int J Mol Sci. 2023 Jan 6;24(2):1122. doi: 10.3390/ijms24021122 (PMC9866400; doi:10.3390/ijms24021122)
Supplement: Supplementary file 1 [file ijms-24-01122-s001.zip › ijms-2125130-supplementary.pdf]

Supporting Information

Upconverting Nanoparticles as a New Bio-Imaging Strategy—Investigating Intracellular Trafficking of Endogenous Processes in Neural Tissue

Karolina Zajdel [1,2,*], Justyna Janowska [1], Małgorzata Frontczak-Baniewicz [1], Joanna Sypecka [1] and Bozena Sikora [2,*]

[1] Mossakowski Medical Research Institute, Polish Academy of Sciences, 5 Pawinskiego Str., 02-106 Warsaw, Poland
[2] Institute of Physics, Polish Academy of Sciences, Al. Lotników 32/46, 02-668 Warsaw, Poland
* Correspondence: kzajdel@imdik.pan.pl (K.Z.); sikorab@ifpan.edu.pl (B.S.)

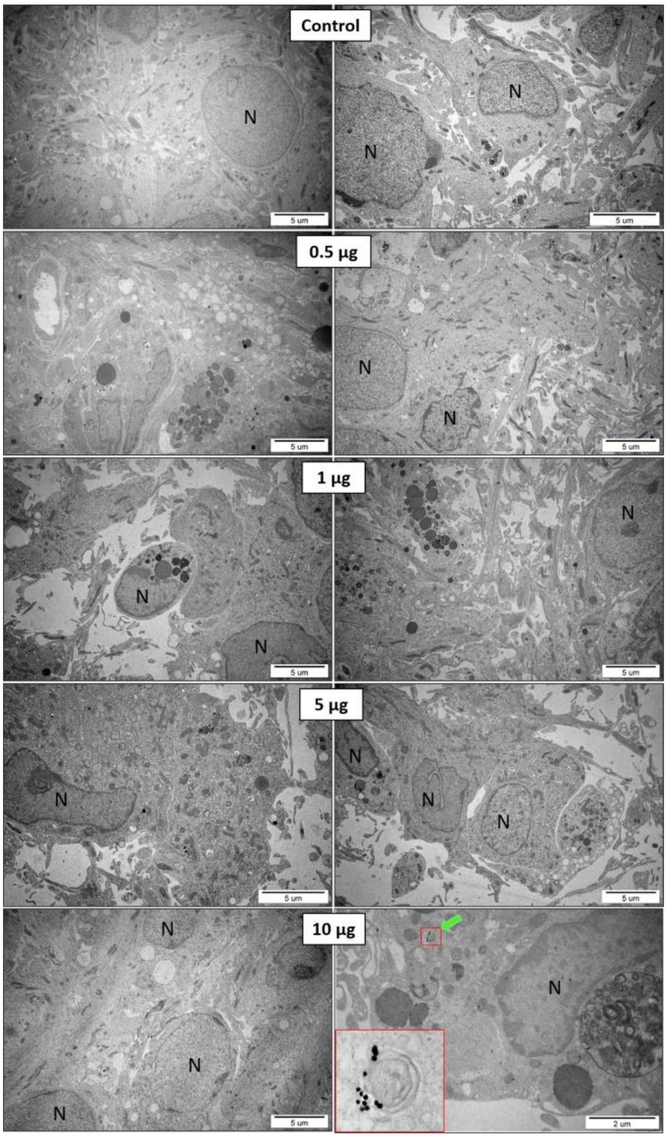

Figure S1. Variant 1. The cells of OHSCs after 24 h of incubation without UCNPs (control) and with 0.5; 1; 5, and 10 µg ml^{-1} of UCNPs. The OHSCs were placed on the membrane. The UCNPs present in the endosome at a 10 µg ml^{-1} concentration are marked with a green arrow (insert with a red outline at higher magnification); N – nucleus.

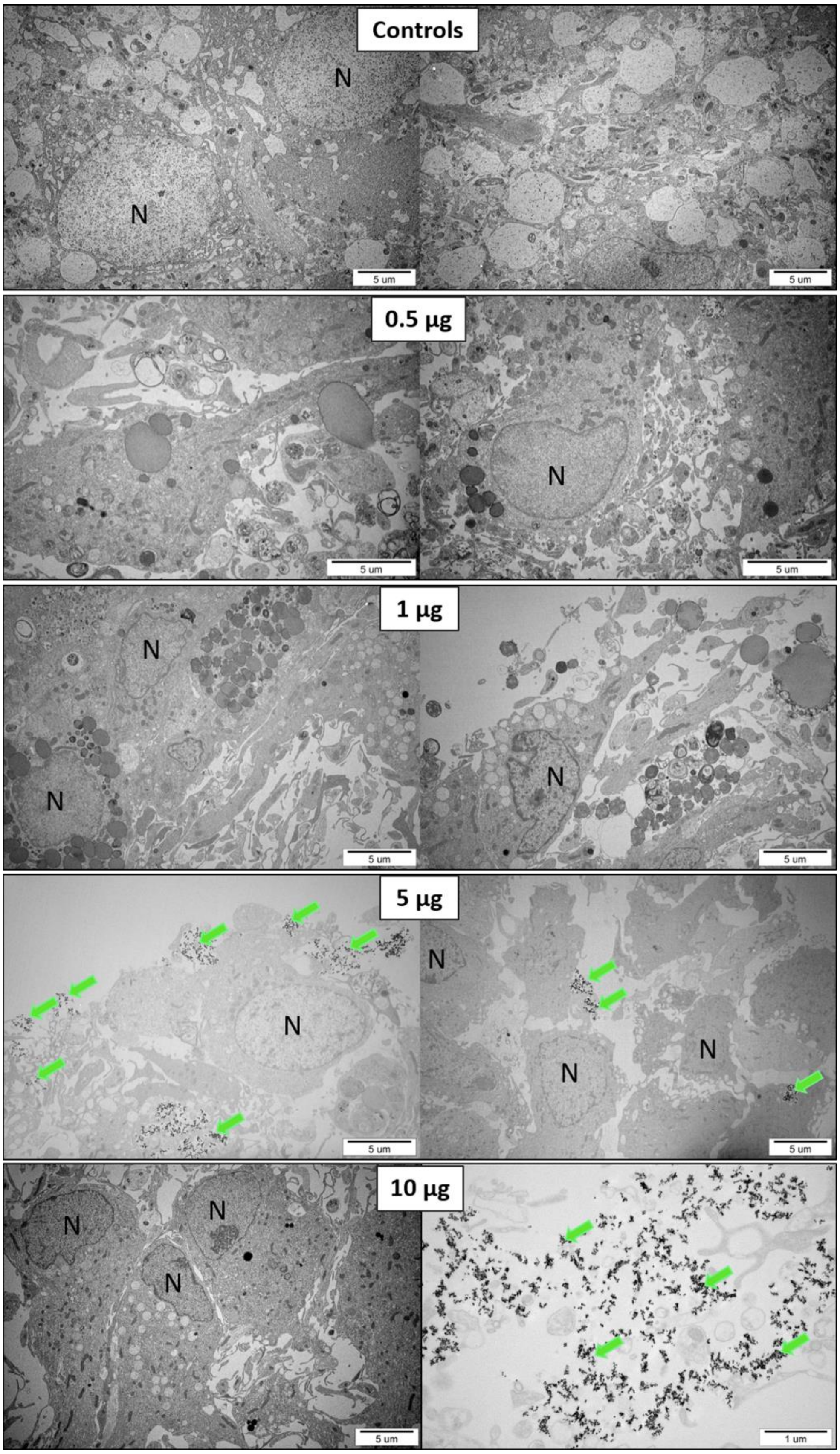

Figure S2. Variant 2. The OHSCs after 1 h incubation without UCNPs (control) and with 0.5; 1; 5, and 10 μg ml^{-1} of UCNPs. The OHSCs were placed on the membrane and immersed in an additional 1 ml of medium. Green arrows mark UCNPs aggregates located outside the cells; N – nucleus.

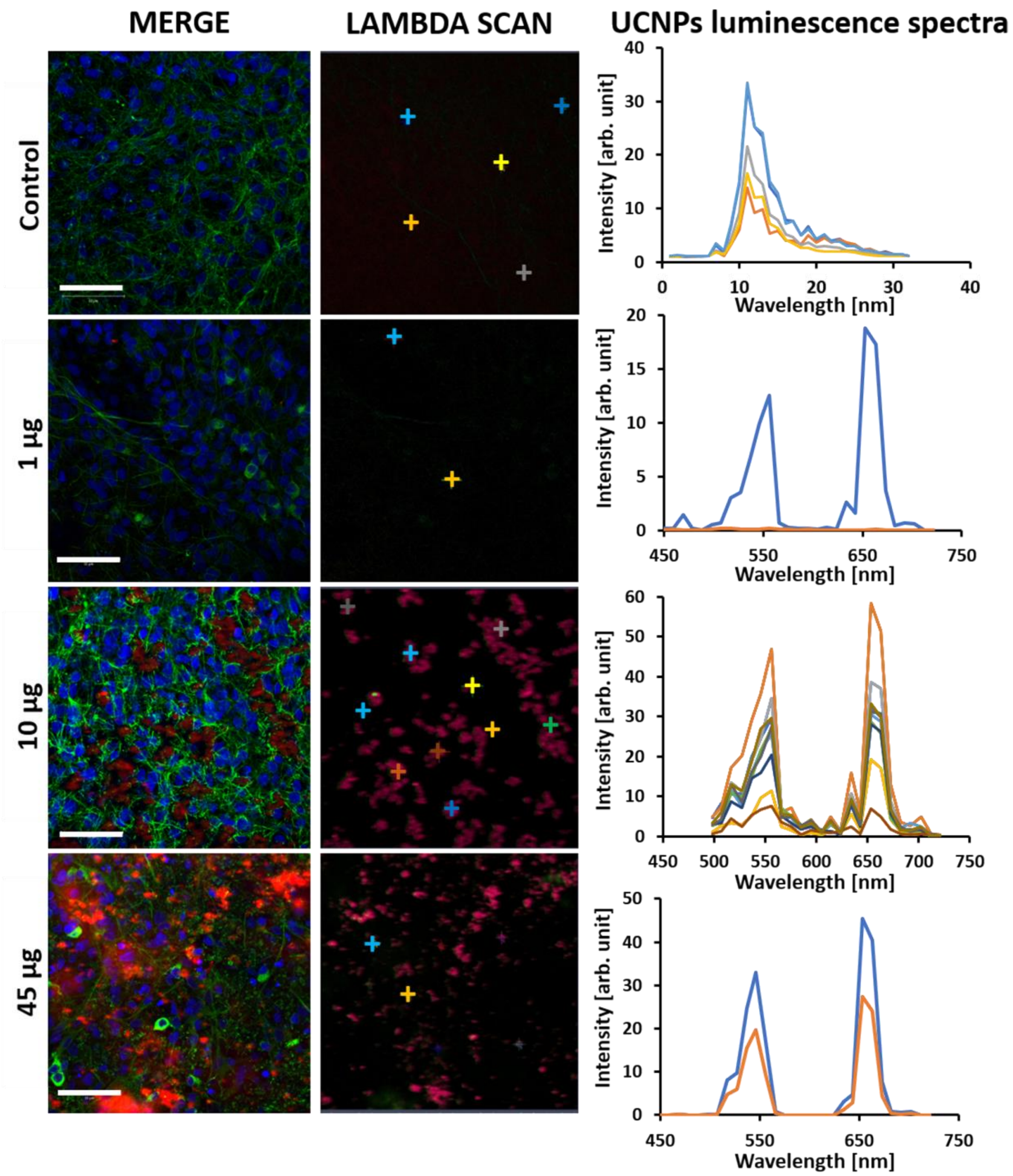

Figure S3. The MERGE confocal images and a highlighted channel for the lambda scan mode and UCNPs luminescence spectra. The red color indicates UCNPs, the green color - neurons (MAP2), and the blue color - cell nuclei. Scale bar - 50 µm

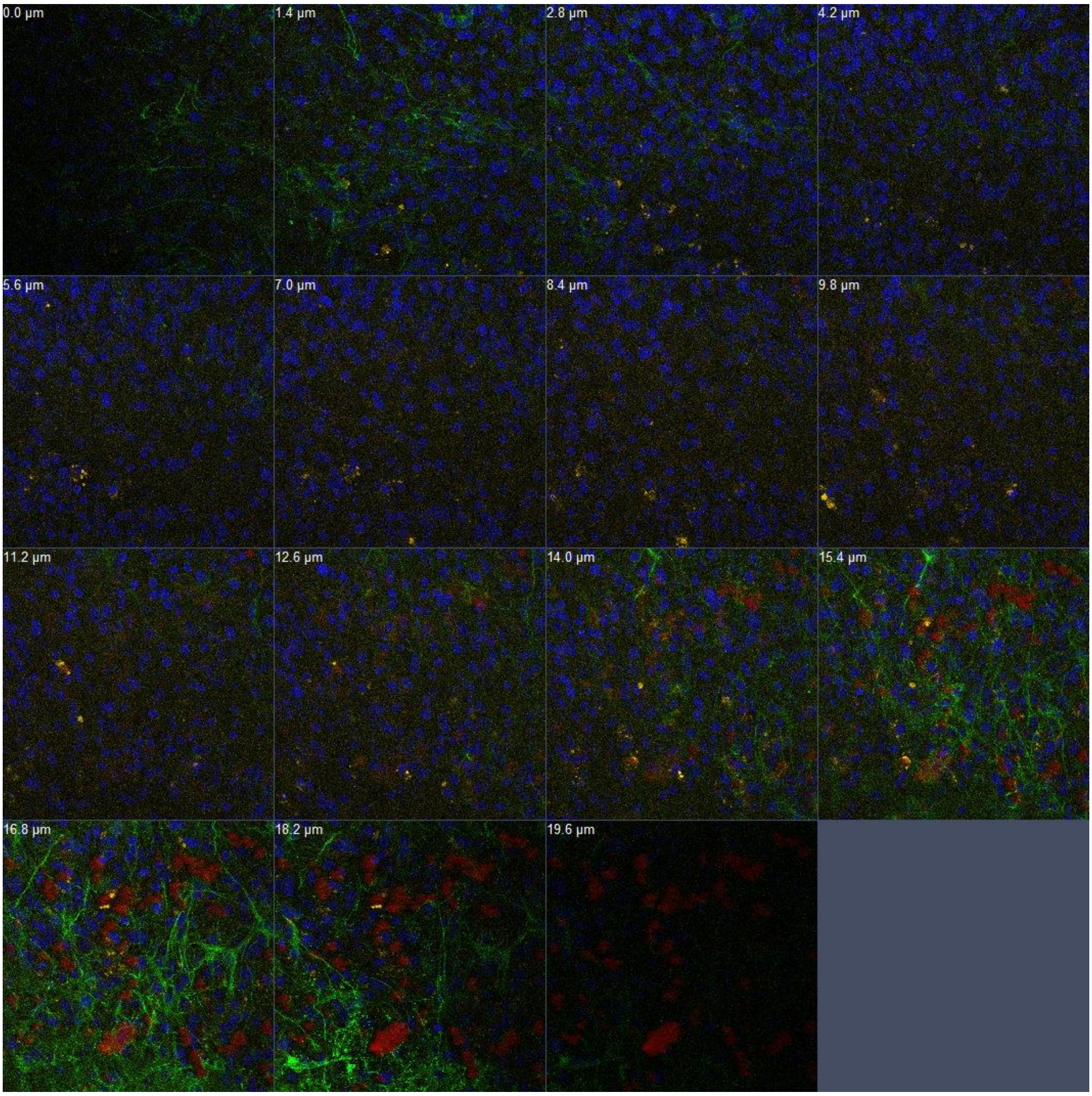

Figure S4. The z-stack analysis of rat hippocampal slices incubated with 1 µg of UCNPs (1 µg in 4 µl) for 24 hours. Neurons are green, cell nuclei are blue, and the luminescence of UCNPs is marked red.

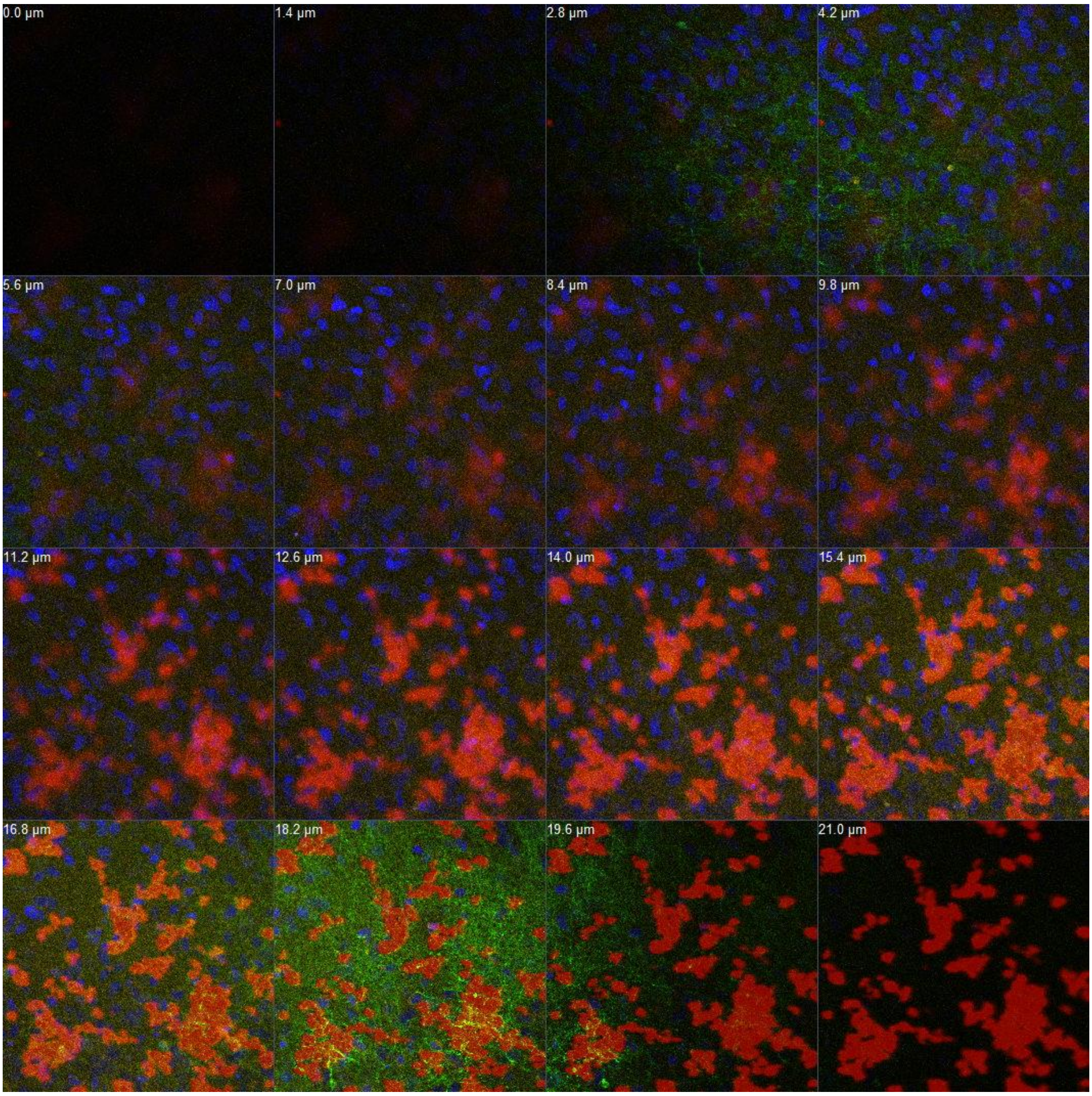

Figure S5. The z-stack analysis of rat hippocampal slices incubated with 10 µg of UCNPs (1 µg in 4 µl) for 24 hours. Neurons are green, cell nuclei are blue, and the UCNPs luminescence is marked by red.

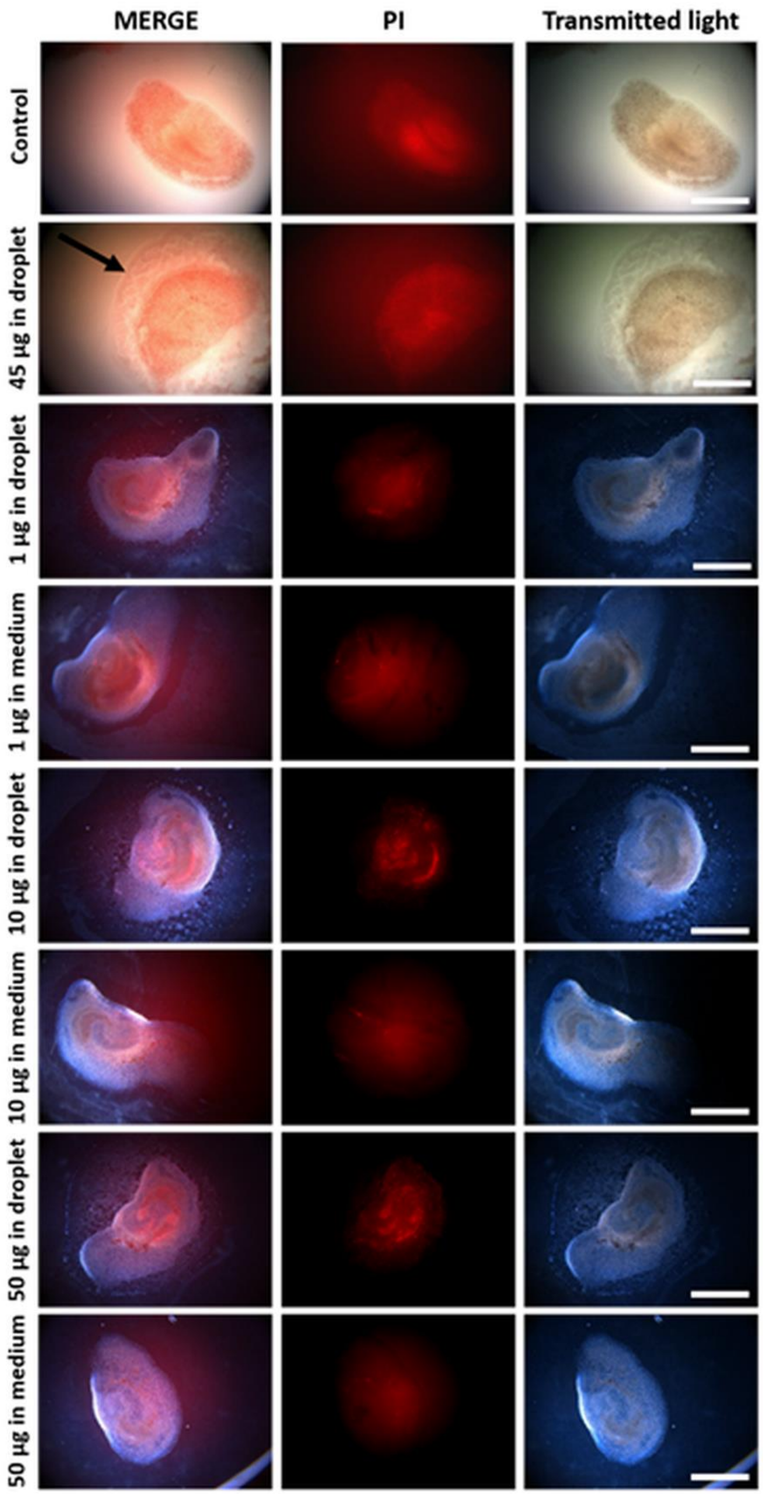

Figure S6. Microscopic visualization of the influence of UCNPs in various concentrations (added directly to the culture medium or applied in the form of droplets directly on the surface of the slices) on the viability of hippocampal cells. The border of the applied droplet is marked with a black arrow. The images were taken at 5x magnification. First channel MERGE - superimposed images, second red channel - PI, third channel - transmitted light of a light microscope. Scale bar - 200 µm.

TEM analysis

After 1-, 2- or 24-hour exposure of OHSC to UCNPs, the slices were gently transferred to 1.5 ml tubes containing fixative (a mixture of 2% PFA and 2.5% GA and in 0.2 M cacodylate buffer pH 7.4) and incubated at 4°C for 24 hours. After this time, the slices were washed three times in 0.1 M cacodylate buffer and post-fixed in a 1% osmium tetroxide for 1 hour. The slices were dehydrated in a series of alcohols with increasing concentration (30%-99.8%) and propylene oxide. Next, the samples were embedded in resin blocks and polymerized at 60°C for 24 hours. The polymerized material was cut into ultra-thin sections (40-60 nm) on an MTXL ultramicrotome (RMC, USA) and placed on copper grids. The double contrast method with uranyl acetate and lead citrate was omitted to exclude the presence of other metal precipitates or artifacts. The accelerating voltage of the microscope during the observation was 80 kV.

Immunohistochemical staining

Immunohistochemical analyses were performed to confirm the presence of UCNPs within the hippocampal cells. The immunohistochemical preparations were imaged using a 710 NLO confocal microscope (Carl Zeiss, Germany) equipped with a femtosecond laser (Coherent, Chameleon).

After 24-hour incubation with the appropriate concentration of UCNPs, slices were washed three times with PBS for 15 min to remove excess UCNPs not internalized by the cells. Slices were then incubated in a freshly prepared 4% paraformaldehyde solution in PBS for 40 min. Subsequently, slides were washed three times in PBS and blocked in 10% goat serum in PBS containing 0.1% Triton X-100 for 1 hour at room temperature. To visualize the UCNPs within various nervous tissue cells, organotypic hippocampal slices were incubated with selected primary antibodies listed in Table 1. Incubation for primary antibodies directed against the MAP-2 microtubule-stabilizing protein and Glial Fibrillary Acidic Protein (GFAP) in 5% goat serum was carried out overnight at 4 °C. Subsequently, the preparations were washed three times with PBS buffer. The slices were incubated for 1-hour with a goat anti-mouse secondary antibody with Alexa Fluor 488 diluted in 5% goat serum. Following incubation, the slices were rewashed three times with PBS buffer. The final step was staining the cell nuclei with a 5 μM solution of Hoechst 33258 dye (1:150 dilution) in PBS for 15 minutes. The slices were washed three times with PBS, mounted on slides, and sealed with a Fluorescence Mounting Medium (Dako) and a coverslip. The prepared preparations were stored at 4 °C.

Table S1. Primary and secondary antibodies used in immunocytochemical studies.

Primary and secondary antibodies	Dilution	Cat No.
Anti-MAP2 (2a+2b) antibody, Mouse monoclonal (Neurons marker)	1:500	Sigma, M1406
Anti-Glial Fibrillary Acidic Protein antibody, Mouse monoclonal (Astrocytes (Macroglia) marker)	1:200	Dako, M0761
Goat anti-Mouse IgG1 Cross-Adsorbed Secondary Antibody, Alexa Fluor™ 488	1:1000	Invitrogen, A21121

Three channels on the confocal microscope of the OHSC with UCNPs were observed. The first channel was used for UCNPs imaging, with excitation at 980 nm (femtosecond laser) and detection at 500-730 nm. The second channel was used for the nucleus labeled with Hoechst 33258 imaging, with excitation at 705 nm (femtosecond laser) and detection in the 425-475 nm range. The third channel was used for neurons or astrocytes labeled with a secondary antibody conjugated to Alexa Fluor 488 imaging, with 488 nm excitation (continuous laser) and detection in the 495-572 nm range.
